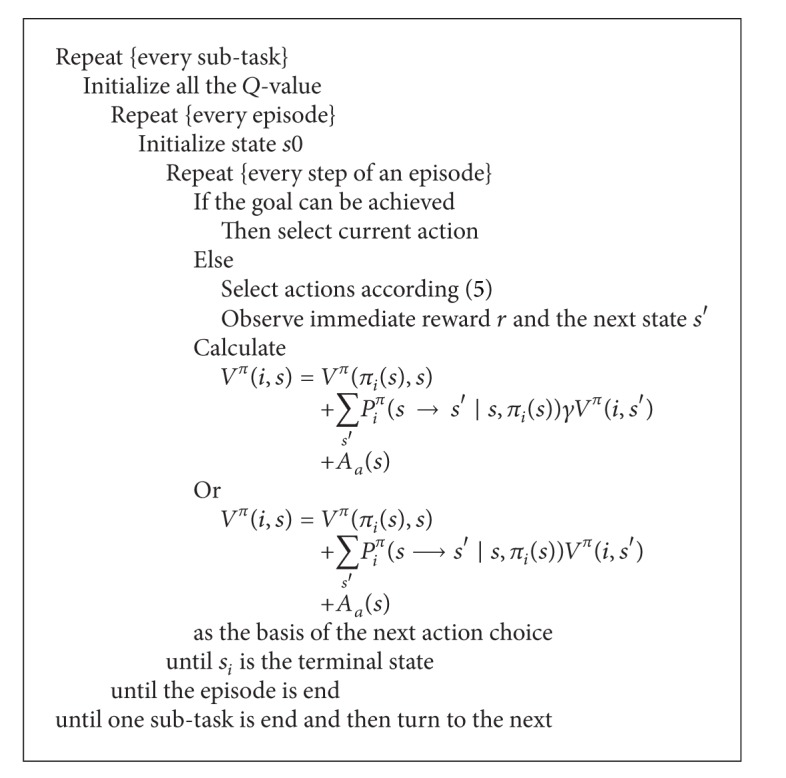# A Reward Optimization Method Based on Action Subrewards in Hierarchical Reinforcement Learning

**DOI:** 10.1155/2014/120760

**Published:** 2014-01-28

**Authors:** Yuchen Fu, Quan Liu, Xionghong Ling, Zhiming Cui

**Affiliations:** ^1^Suzhou Industrial Park Institute of Services Outsourcing, Suzhou, Jiangsu 215123, China; ^2^School of Computer Science and Technology, Soochow University, Suzhou, Jiangsu 215006, China

## Abstract

Reinforcement learning (RL) is one kind of interactive learning methods. Its main characteristics are “trial and error” and “related reward.” A hierarchical reinforcement learning method based on action subrewards is proposed to solve the problem of “curse of dimensionality,” which means that the states space will grow exponentially in the number of features and low convergence speed. The method can reduce state spaces greatly and choose actions with favorable purpose and efficiency so as to optimize reward function and enhance convergence speed. Apply it to the online learning in Tetris game, and the experiment result shows that the convergence speed of this algorithm can be enhanced evidently based on the new method which combines hierarchical reinforcement learning algorithm and action subrewards. The “curse of dimensionality” problem is also solved to a certain extent with hierarchical method. All the performance with different parameters is compared and analyzed as well.

## 1. Introduction

Reinforcement learning (RL) is one kind of interactive learning methods. Its main characteristics are “trial and error” and “related reward.” In the whole learning process, the agent can communicate with environment to get rewards and improves actions according to these rewards. Repeat the process over enough times, an optimal action can be gained, and all the optimal actions compose optimal policies.

Q-learning, which tries to map every state-action pair into a real number (Q-value), is one of the basic and classical methods in reinforcement learning field. But it is only suitable for small state spaces. When state spaces become larger or even infinite and continuous, it is not effective to visit and store the same states time after time. Then there arose the “curse of dimensionality” problem. Recently, the main ideas used to solve this problem are abstraction and approximation. All these ideas can be divided into four kinds of methods: spaces clustering algorithms, finite spaces search methods, value functions approximation methods, and hierarchical reinforcement learning (HRL) [1]. A reinforcement learning method based on node-growing k-means clustering algorithm is described in the literature [2], which is used to solve the adaptive partition problem in continuous state spaces. There are also many function estimate methods in value function approximation, such as neural network and kernel methods [3, 4]. The relational reinforcement learning (RRL) method, which had been researched and expatiated in the literature [5–7], has been concerned most in recent years. In order to get a further purpose, other methods such as Gaussian processes, graphical kernels, and logical algorithm had been combined with RRL as well [8–10]. At present, the achievements of HRL method include Option [11], MAXQ [12], and HAM [13]. These methods decompose the learning task into a hierarchy of smaller subtask. Thus, in HRL method, if the agent is only concerned about the current local space change or the subtarget state change, the update process of the policy will be restricted in the local space or high-level space, so that you can speed up the learning process and reduce the dependence of the algorithm's convergence speed with the environmental changes.

At present, the Option and MAXQ of the HRL algorithms have been widely used. They have proved that they not only can be applied to the task which has huge state space, but also can apply the experience to other similar learning tasks, greatly accelerating the learning speed. Schultink et al. proposed an EHQ algorithm based with MAXQ. This algorithm is, in theory, able to converge to the hierarchical optimal, compared with MAXQ which can only converge to the recursively optimal policy [14]. To use the HRL method, we must first solve the two problems: (1) search the subgoal of the subtask and (2) decompose the task automatically. Many ways have been offered to the search for the subgoal. All these methods are based on the experience gained in the past to get the subgoals, which can be divided into two types; one is based on the statistical characteristics of the state access frequency to find subgoals.

Representative achievements are as follows: Mannor et al. think that states serve as potential subgoals if they are frequently visited on successful paths but are not on unsuccessful visited ones [15]; Stolle and Precup use the nodes visited most frequently as a subgoal [16]. In these two approaches, sometimes the results obtained are not very satisfactory, because the state which is around the real subgoals state may also be visited many times; Şimşek et al. find the subgoals by dividing the recent experience of local state transition diagram [17]; Subgoals' finding is ultimately server for decomposing the task automatically.

Because of the “curse of dimensionality” problem in most reinforcement learning tasks and the low convergence speed of traditional algorithms, it is important to improve both of them in RL methods. The key problems solved in this paper are “curse of dimensionality” problems in large discrete state spaces of infinite repetition tasks and the reward functions optimization in convergence speed enhancement of algorithms such as Q-learning. At the same time, action subrewards are proposed to evaluate the efficiency of actions for achieving the goal. Combined with a hierarchical reinforcement learning method in large discrete state spaces of infinite repetition tasks, it is applied to the Tetris game as an experiment. The experiment result shows that both of the problems mentioned above can be solved to a certain extent.

## 2. Reinforcement Learning

**Figure 1:**
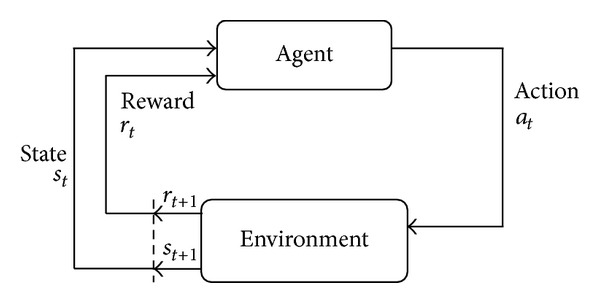
The framework of reinforcement learning.

Reinforcement learning is a concept that parallels with supervised learning and unsupervised learning. The main difference between them is that, in RL, the agent is not told by positive and negative examples, but it uses trial-and-error method to find optimal policies instead. The whole learning process is evolved from ideas in psychology. The agent gets rewards form the interaction with its environment so as to enhance or weaken execution of actions for getting the optimal policies. So the final purpose of reinforcement learning is to maximize the accumulative reward, and the learning framework is showed in Figure 1.

### 2.1. Markov Decision Processes (MDP)

There exist many kinds of modeling methods for environment in reinforcement learning. For stochastic, discrete states and discrete time tasks, it used to take the Markov model. The task in this paper is with discrete states and time, and the states space is huge and stochastic, so the learning process can be modeled as Markov decision processes (MDP). There gives the formalized definition [18, 19]. 

*Markov Decision Processes.* It is defined by a tuple 〈*S*, *A*, *R*, *P*〉, which contains an environment states set *S*, an actions set *A*, a reward function: *R* : *S* × *A* → *R*, and a state transition function: *P* : *S* × *A* → *PD*(*S*′). *R*(*s*, *a*, *s*′) is the immediate reward in state *s* under action *a* transited to state *s*′. *P*(*s*, *a*, *s*′) is the transition probability in state *s* under action *a* to state *s*′. Laconically, both of the variables can be written as *R*_*ss*′_^*a*^ and *P*_*ss*′_^*a*^.

Based on the above definition, the key work of reinforcement learning is to solve learning problem with unknown *R*_*ss*′_^*a*^ and *P*_*ss*′_^*a*^. So the only experience we can use is immediate rewards, which are contacted with state value functions and policies to choose the right policy. And it can be gained that

(1) $$\begin{array}{c} R_{t}=r_{t+1}+\gamma r_{t+2}+\gamma^{2}r_{t+3}+\cdots =r_{t+1}+R_{t+1}. \end{array}$$

So, for the special policy, the Bellman equation is as follows:

(2) Vπ(s)=Eπ{Rt ∣ st=s}=Eπ{∑k=0∞γkrt+k+1 ∣ st=s}  =Eπ{rt+1+γ∑k=0∞γkrt+k+2 ∣ st=s}  =∑aπ(s,a)∑s′Pss′a[Rss′a+γEπ×{∑k=0∞γkrt+k+2 ∣ st+1=s′}]=∑aπ(s,a)∑s′Pss′a[Rss′a+γVπ(s′)].

According to the optimal Bellman equation, the value function of optimal policy is defined as

(3) V∗(s)=max⁡a∈A(s) E{rt+1+γV∗(st+1) ∣ st=s,at=a}=max⁡a∈A(s) ∑s′Pss′a[Rss′a+γV∗(s′)].

Solve the above Bellman equation, and we can get the solution for MDP.

### 2.2. Hierarchical Reinforcement Learning

The hierarchical reinforcement learning (HRL) is proposed by many researchers to solve the “curse of dimensionality” problem. Its essential is adding abstraction to the reinforcement learning framework and dividing a whole task into many different subtasks on different hierarchy, which makes it possible to solve the task in smaller substate spaces, and, as a result, the convergence speed can be enhanced as well. From another point of view, this can be seen as a method for dimensionality reducing, which is good for the solution of problems. Main techniques for HRL include state space decomposition, temporal abstraction, and state abstraction. Classical algorithms are Option, HAM and MAXQ, which considered the hierarchy from angles of action, policy, and task [1]. These are methods researched and applied widely in recent years.

State space decomposition is a method which decomposes the state space into different subsets and then gets solutions with the divide and rule policy. As a result, all the problems can be resolved in small subspaces. Temporal abstraction is a method that groups action sequences and action sets, which divides a single step into many steps so as to decrease the decision-making number and reduce the learning press. State abstraction method, which ignores variables that are irrelative to subtasks, achieves the effect of dimension reduction.

## 3. Function Optimization Based on Subrewards in Hierarchical RL

### 3.1. A Hierarchical Method in Infinite Repetition Tasks

On the basis of the theory about reinforcement learning and HRL mentioned in Section 2, a hierarchical method in infinite repetition tasks is proposed. According to the characteristic of tasks in this paper, the state space is decomposed to reduce dimensionality and speed up convergence.

The infinite repetition task *M* can be decomposed into a task set {*M*_0_, *M*_1_,…, *M*_*n*_}, and because of the repetition of the task, the policy *π* can be decomposed into a policy set {*π*_0_, *π*_1_,…, *π*_*n*_}, where *π*_*i*_ is the corresponding policy to *M*_*i*_ and each *π*_*i*_ is parallel. Then the set of each optimal policy *π*_i_* in subtask *M*_*i*_ is π* = {*π*_0_*, *π*_1_*,…, *π*_n_*} which is an optimal policy of the whole task. To some extent, all the subtasks are independent of each other.

Let a subtask *M*_*i*_ be defined by a couple 〈*π*_*i*_, *R*_*i*_〉, where *π*_*i*_ is a policy for a subtask and *R*_*i*_ is a reward for a subtask. Let *V* ^*π*^(*i*, *s*) be the expect reward in subtask *M*_*i*_ with policy *π*_*i*_, and the corresponding Bellman equation is as follows:

(4) Vπ(i,s)=Vπ(πi(s),s) +∑s′Piπ(s⟶s′ ∣ s,πi(s))γVπ(i,s′).

The hierarchical idea in the infinite repetition task is much more compact than that in the MAXQ method. Because of the repetition, it is easy for us to handle states and actions, and policies can be utilized time after time as well.

### 3.2. Action Subreward

The exploration and exploitation of actions are always key problems in reinforcement learning, and they are also important points in the solution of convergence speed. Traditional RL methods such as Q-learning could not find a balance between them, and there also exists the low convergence speed problem; that is, the agent has to visit the same states over and over again to achieve the goal for rewards. The larger the state spaces become, the lower the convergence speed would be. There needs to be some efficient way to optimize the reward functions to handle these problems and enhance the convergence speed.

A method based on subrewards is proposed in this paper, which considers the efficiency of actions along with their rewards. The estimate standard is whether actions are good for goal achieving or not. It makes for action choice and resolves the balance between action exploration and exploitation as well. If state values weigh the action quality in current state, then the action subrewards stand for the detailed effect of the action in current state. All these make the learning algorithm more flexible, optimize reward functions, and enhance convergence speed.

**Definition 1**

The action subreward *A*_*a*_(*s*) denotes the efficiency value for agent implements action *a* to achieve goal in state *s*, instead of the direct reward it gets.

For a special task, let characteristic set *T* that affects goal achieving be {*t*_0_, *t*_1_,…, *t*_*n*_}, and then the action subreward in state *s* with action *a* in that task can be represented as follows:

(5) $$\begin{array}{c} A_{a}\left(s\right)=\alpha_{0}t_{0}+\alpha_{1}t_{1}+\cdots +\alpha_{n}t_{n}=\sum_{t_{i}\in T}\alpha_{i}t_{i}, \end{array}$$

where *α*_*i*_ (−1 ≤ *α*_*i*_ ≤ 1) is the proportion parameter of each characteristic, and it can be confirmed by its effect in goal achieving. It can be a prize which is good for goal achieving or a punishment that gets the opposite effect.

### 3.3. Reward Function Optimization Based on Action Subrewards in Infinite Repetition Tasks

According to the theory mentioned in Sections 3.1 and 3.2, the reward function optimization based on action subrewards in infinite repetition tasks can be confirmed now, which means that some modification can be done to (4). It should be divided into two aspects:

(1) there are still many states in subtasks, in which discount reward model is adopted, and its equation can be modified as
  (6) Vπ(i,s)=Vπ(πi(s),s) +∑s′Piπ(s⟶s′ ∣ s,πi(s))γVπ(i,s′)+Aa(s)=Vπ(πi(s),s) +∑s′Piπ(s⟶s′ ∣ s,πi(s))γVπ(i,s′)+∑ti∈Tαiti;
(2) there are a few states in subtasks, in which finite-horizon model is taken, and the equation can be modified as
  (7) Vπ(i,s)=Vπ(πi(s),s) +∑s′Piπ(s⟶s′ ∣ s,πi(s))Vπ(i,s′)+Aa(s)=Vπ(πi(s),s) +∑s′Piπ(s⟶s′ ∣ s,πi(s))Vπ(i,s′)+∑ti∈Tαiti.

From (6) and (7), it can be concluded that if actions *a*_*i*_ and *a*_*j*_ are taken in a special state, the rewards got from them will be indistinctive in nonterminal states or similar states. This needs the agent to balance the exploitation and exploration of actions, which will spend too much time and calculation to finish this work. But with action subrewards, *a*_*i*_ and *a*_*j*_ can be evaluated in detail to get further rewards and make reasonable decisions. At the same time, all the proportion parameters can be adjusted from a microcosmic angle, and it is more flexible for action choice control.

### 3.4. Reward Function Optimization Algorithm

On the basis of the theory discussed above, the framework of reward function optimization algorithm is as in Algorithm 1.

For the convergence of this algorithm, what we need to discuss is only the posterior part of (6) and (7), because the *V* ^*π*^(*π*_*i*_(*s*), *s*) + ∑_*s*′_ *P*_*i*_^*π*^(*s* → *s*′ | *s*, *π*_*i*_(*s*))*V* ^*π*^(*i*, *s*′) part has been proved to be convergent in other literature (please turn to interrelated papers).

**Proof**

Let Δ be the most error between *V* ^*π*^(*i*, *s*) and *V* ^*π*^(*i*, *s*′); that is,

(8) $$\begin{array}{c} \Delta =\max\left|V^{\pi }\left(i,s^{\prime }\right)-V^{\pi }\left(i,s\right)\right|. \end{array}$$

Because −1 ≤ *α*_*i*_ ≤ 1 in ∑_*t*_*i*_∈*T*_ *α*_*i*_ *t*_*i*_, whether it is *α*_*i*_ ≥ 0 or *α*_*i*_ ≤ 0, that will be good for choosing the optimal actions, and the speed of Δ → 0 is enhanced; that is, the ∑_*t*_*i*_∈*T*_ *α*_*i*_ *t*_*i*_ part quickens the convergence speed.

The proof of (7) is the same as (6), and it will not give unnecessary details here.

### 3.5. Action Subrewards with Divide and Rule

In order to increase flexibility of the algorithm, considering the changes in different periods, the idea of divide and rule is introduced in this paper. It is rooted in hierarchical method. Because of the diversification in tasks, although there is only one goal in a task, factors that impact goal achieving will change a lot in different periods, which changes the efficiency of action subrewards as well. And then the idea of divide and rule is introduced. It is reasonable and flexible to adjust proportion parameters in time for action selecting when the environment changes. This kind of idea is similar to the hierarchical concept, and it can be understood as a hierarchical method on the action subrewards.

This theory is validated by the experiment in this paper. The result shows that the performance can be improved with such a method.

## 4. Experiment and Analyses

The Tetris game was developed by Alexey Pathitov in 1985. Because of its characteristic, it has been a classical problem for large discrete state spaces in reinforcement learning. At the same time, the Tetris game is an infinite repetition task, which needs the player to keep the game continuing and get as many scores as possible. All these factors are the reason why we took the Tetris game as our experiment. In a standard game platform, the game ground is a matrix with 10 columns and 20 rows, there are seven kinds of blocks, most of which have four directions, and the number of states will be 10^60^, which is a large discrete state space problem for both store and calculation to computers. The Tetris game is exactly a typical representation of the “curse of dimensionality” problem.

The Tetris game is an online game that has high demands upon calculating speed and convergence speed. All of that can be handled with methods proposed in this paper. In the experiment, a standard Tetris game platform is selected (i.e., a 10 × 20 matrix and seven kinds of blocks), and we can gain 10 points after one line is filled up. All the points we get are nonspecial; that is, the points got from lines filled up one by one are equal to as many lines filled up once. The action set contains turn left, turn right, circumrotate, and go down. It should be noted that because of the small number of states in subtasks after hierarchical handling, the finite-horizon model is employed here. The set of characteristics that affect goal achieving (it is get scores in this experiment) is *T* = {height, hole, channel}, and the corresponding proportion parameters are *α*, *β*, and *γ*, which are counterparts of *α*_1_, *α*_2_, and *α*_3_ in (5).

**Figure 2:**
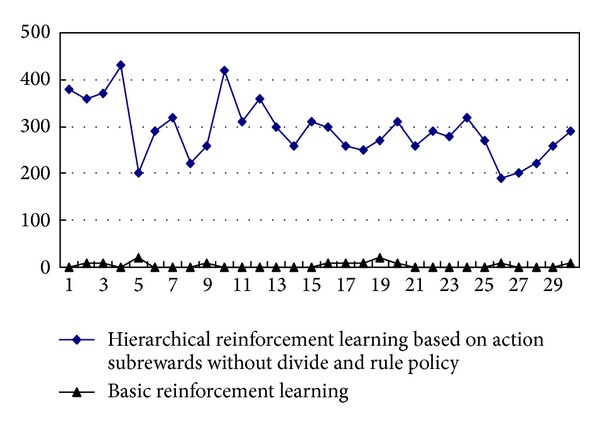
The performance comparison between hierarchical reinforcement learning based on action subrewards without divide and rule policy and basic reinforcement learning.

Figure 2 shows the performance compared, between hierarchical reinforcement learning based on action subrewards without divide and rule policy and basic reinforcement learning, where proportion parameters are set as *α* = 0.1, *β* = 0.2, *γ* = 0.1. The *y*-axis denotes the number of lines filled up, and the abscissa denotes the number of training steps.

It is easy to conclude that the learning efficiency and outcome of hierarchical RL based on action subrewards are much better than that of the basic RL method. It is obvious that the basic RL method is hard to converge because of the large state spaces in most instances. But after combined action subrewards and hierarchical method, the scores increase obviously. The real-time require is satisfied as well. Even if there are no prior knowledge and training, it works well.

**Figure 3:**
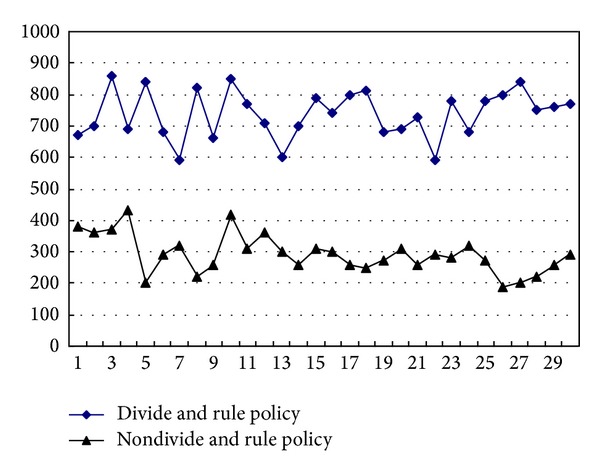
The performance comparison between divide and rule policy and nondivide and rule policy.

The divide and rule policy that adjusts proportion parameters of *α*, *β*, and *γ* is introduced in this experiment to make the learning more flexible, which makes action subrewards more reasonable. And then action subrewards can reflect action quality in time.  Figure 3 shows the performance comparison between divide and rule policy and nondivide and rule policy, where exist two situations according to the height of blocks: *α* = 0.1, *β* = 0.2, and *γ* = 0.1 as well as *α* = 0.2, *β* = 0.1, and *γ* = 1; that is, when the height is low, holes could be considered more than height, and height should be considered more with it growing. So policy changes with the environment, which is according to the reality. The result shows that the performance of algorithm improves a lot and scores are much higher.

## 5. Conclusions

Reinforcement learning which is a method that is independent of supervised learning or unsupervised learning has been studied and applied widely. But there is “curse of dimensionality” problem in basic RL methods, and it is so hard to avoid and conquer, so many solutions had been proposed to resolve this kind of problem.

Aiming at solving “curse of dimensionality” problem and the low convergence speed in RL, a hierarchical RL method based on action subrewards is proposed. Apply it to the Tetris game, and the outcome shows that it is efficient in getting over not only the “curse of dimensionality” problem but also the difficulty of low convergence speed. The whole effect of the experiment has been improved much as well.

But there would be some local optimization problem in this experiment because of the hierarchical method. And we need to do further research on the choice of proportion parameters to make sure the algorithm is more efficient. Both of the problems mentioned above will be our key work in the future, and there are still many things we have to do to make the result closer to the global optimization and improve the performance of algorithm.

## Figures and Tables

**Algorithm 1:**